# Impaired Left Atrial Function in Patients with Embolic Stroke of Undetermined Source

**DOI:** 10.3390/jcm15197378

**Published:** 2026-09-23

**Authors:** Urszula Gancarczyk, Andrea Stefanini, Paweł Prochownik, Marcin Jakiel, Michał Tworek, Wojciech Płazak, Matteo Cameli, Monika Komar

**Affiliations:** 1 Department of Transplantology and Mechanical Circulatory Support, Krakow Specialist Hospital Named After St. John Paul II, ul. Prądnicka 80, 31-202 Kraków, Poland; 2 Doctoral School of Medical and Health Sciences, Jagiellonian University Medical College, ul. Św. Łazarza 16, 31-530 Kraków, Poland; 3 Department of Medical Biotechnologies, Division of Cardiology, University of Siena, Viale Mario Bracci 16, 53100 Siena, Italy; astefanini94@gmail.com (A.S.);; 4 Department of Cardiac and Vascular Diseases, Institute of Cardiology, Jagiellonian University Medical College, ul. Prądnicka 80, 31-202 Kraków, Poland

**Keywords:** embolic stroke of undetermined source (ESUS), left atrial strain (LAS), atrial cardiomyopathy

## Abstract

**Background/Objectives:** Embolic Stroke of Undetermined Source (ESUS) refers to non-lacunar ischemic stroke without an identified cause and is presumed to be related to a cardioembolic mechanism. A potential etiological factor is atrial fibrosis leading to left atrial (LA) dysfunction, also known as atrial cardiomyopathy. This study aimed to compare advanced transthoracic echocardiographic parameters of LA function in patients with ESUS and age- and sex-matched controls. **Methods:** In this prospective matched observational case–control study, fifty patients with ESUS and 50 age- and sex-matched controls were analyzed. All participants underwent comprehensive transthoracic echocardiography (TTE). LA parameters included four-dimensional volumetry, LA strain (LAS), and LA stiffness index calculated as the ratio of E/eʹ to reservoir strain. All ESUS patients additionally underwent transesophageal echocardiography (TEE) to assess the presence of patent foramen ovale (PFO). **Results:** Compared with controls, ESUS patients had significantly reduced LAS in the reservoir phase (LAS-r 37.3% vs. 42.3%; *p* < 0.01) and conduit phase (LAS-cd 22.9% vs. 28.4%; *p* < 0.01), as well as a higher LA stiffness index (0.171 vs. 0.149; *p* < 0.01). No significant differences were found in LA size or volume. In subgroup analysis, ESUS patients with and without PFO did not differ, while both subgroups showed significant impairment compared with controls. **Conclusions:** ESUS is associated with impaired LA functional properties despite preserved LA size. LAS may provide complementary information on subclinical LA functional abnormalities in patients with ESUS; however, its clinical and prognostic significance requires validation in prospective studies.

## 1. Introduction

Embolic Stroke of Undetermined Source (ESUS) accounts for approximately 17% of all ischemic strokes. According to the classification proposed in 2014, ESUS is defined as a non-lacunar stroke in which no clear cause is identified after a standard diagnostic workup. Specifically, significant carotid artery atherosclerosis, occlusion of intracranial arteries, supraventricular arrhythmias such as atrial fibrillation or flutter, and major-risk cardioembolic sources must be ruled out. Compared to the general population of patients with ischemic stroke, ESUS patients tend to be younger, present with smaller infarct size, and have a higher risk of recurrent stroke [1,2,3].

Potential causes of ischemia in ESUS patients include cardiac shunts and abnormalities in the structure and function of the left atrium and ventricle, which may predispose to thromboembolism [4,5,6]. Atrial cardiomyopathy, defined as structural, functional, or electrical abnormalities of the atria (e.g., due to fibrotic remodeling), is one proposed mechanism that may lead to thromboembolic events [7].

Transthoracic echocardiography (TTE) is routinely performed in all patients during the diagnostic work-up for ischemic stroke. However, basic measurements of LA size and volume may be insufficient to identify atrial cardiomyopathy. Speckle-tracking echocardiography enables detection of early functional changes in the left atrium and may be a valuable tool for identifying potential causes of stroke [8,9,10]. In particular, LA strain (LAS), which is correlated with the extent of LA wall fibrosis, may indicate an early phase of atrial cardiomyopathy and serves as a predictor of new-onset atrial fibrillation, even in individuals with normal LA volumes [11,12].

However, data on left atrial function in patients with ESUS remain limited, especially in those with preserved conventional echocardiographic parameters. We hypothesized that patients with ESUS have impaired LA function. Therefore, the aim of our study was to evaluate echocardiographic parameters of LA morphology and function in patients with ESUS compared with age- and sex-matched healthy controls.

## 2. Methods

### 2.1. Study Population

This prospective matched observational case–control study included 50 consecutive patients with ESUS and 50 age- and sex-matched controls. Participants were recruited between April 2022 and December 2024. Patients with ESUS were further stratified according to the presence of patent foramen ovale (PFO).

All patients underwent a standardized diagnostic work-up according to current ESUS diagnostic criteria to exclude alternative causes of cerebral ischemia. Brain magnetic resonance imaging (MRI), transthoracic echocardiography (TTE), and transesophageal echocardiography (TEE) were performed in all patients. The evaluation included 72 h Holter electrocardiogram monitoring to exclude atrial fibrillation or flutter and carotid and vertebral artery ultrasound to exclude ≥50% extracranial arterial stenosis. Patients with known coagulation disorders or thrombophilia were excluded. Patients with moderate-to-severe valvular heart disease were also excluded. The presence of mitral stenosis or more than mild mitral regurgitation was specifically assessed.

The control group consisted of healthy volunteers matched for age and sex, with no history of stroke, cardiovascular disease, or atrial fibrillation.

All study procedures were conducted in accordance with the Declaration of Helsinki. The study protocol was approved by the Jagiellonian University Medical College Bioethics Committee (No. 1072.6120.55.2021; approval date: 23 March 2022), and written informed consent was obtained from all participants.

### 2.2. Echocardiographic Examination

Echocardiographic examinations (TTE and TEE) were performed within 1–3 weeks after the stroke in the study group using the EPIQ 7 system (Philips, Andover, MA, USA), in accordance with the guidelines of the European Society of Cardiology and the American Society of Echocardiography [13,14,15]. In the control group, TTE was performed using the same imaging system and standardized protocol, following the same methodological principles and guideline recommendations.

The LA parameters assessed by transthoracic echocardiography included anteroposterior diameter (parasternal long axis), area, volume, and volume index (biplane Simpson). The LV measurements included end-diastolic diameters, interventricular septum and posterior wall thickness, LV mass, and LV mass index. Systolic function was evaluated using Simpson’s method to calculate ejection fraction. Diastolic function was assessed using pulsed-wave Doppler of mitral inflow (E and A wave velocities, E/A ratio) and tissue Doppler imaging of the lateral and septal mitral annulus (e’ velocities, averaged as e’mean), with calculated E/e’ ratio. Tricuspid regurgitation maximal velocity (TR Vmax) was measured by continuous-wave Doppler. LAS was assessed from a non-foreshortened apical four-chamber view using vendor-specific speckle-tracking software (QLAB version 13.0, Philips Medical Systems, Andover, MA, USA). The QRS complex was used as the reference point for strain analysis. The LA endocardial border was manually traced by a single experienced and independent echocardiographer, delineating a region of interest (ROI) comprising six segments. Manual adjustments of the ROI were performed when necessary. The intra-observer intraclass correlation coefficient (ICC) exceeded 0.90 for all analyzed parameters. Parameters included reservoir strain (LAS-r), conduit strain (LAS-cd), and contraction strain (LAS-ct), expressed as percentage change in myocardial fiber length (Figure 1). LA stiffness index was defined as E/e’ divided by LAS-r. Patent foramen ovale (PFO) was evaluated using transesophageal echocardiography in accordance with current guidelines.

### 2.3. Statistical Analysis

Continuous variables were expressed as mean ± standard deviation (SD) for normally distributed variables, or as medians with interquartile range (IQR) for non-normal distributions. Group comparisons were made using Student’s *t*-test or Mann–Whitney U test. Subgroup comparisons were performed using one-way ANOVA or Kruskal–Wallis test, as appropriate. Receiver operating characteristic (ROC) curve analysis was used to assess the ability of echocardiographic parameters of LA function to distinguish patients with ESUS from healthy controls. The area under the curve (AUC) with 95% confidence intervals (CI) was calculated. Statistical analysis was performed using PS Imago Pro 10 (Predictive Solutions). A *p*-value < 0.05 was considered statistically significant. The sample size was based on the number of eligible patients available during the study period; no formal a priori power calculation was performed.

To minimize potential sources of bias, consecutive patients were enrolled, standardized imaging protocols were used, and echocardiographic measurements were performed by a single experienced observer blinded to clinical data.

## 3. Results

The study included 50 patients (31 women, 19 men), aged 30–54 years (median 42; IQR: 36–50), with ESUS and no evidence of significant cervical artery atherosclerosis, atrial fibrillation or flutter, or coagulation disorders. The control group consisted of age- and sex-matched healthy volunteers. The two groups did not differ significantly in anthropometric parameters including weight, height, body mass index (BMI), or body surface area (BSA) (Table 1).

No significant differences were observed in LV size or in systolic function between groups. ESUS patients showed lower values in parameters associated with diastolic function: E-wave velocity (76.5 cm/s vs. 84.8 cm/s, *p* < 0.05), septal e’ (10.3 vs. 12.2 cm/s, *p* < 0.05), lateral e’ (14.3 vs. 16.0 cm/s, *p* < 0.05), and E/A ratio (1.11 vs. 1.24, *p* < 0.05) (Table 2 section A). There were no significant differences in LA size and volume parameters (Table 2, section B).

In contrast, LA functional parameters showed substantial differences: the study group had a higher LA stiffness index (0.171 vs. 0.149, *p* < 0.05), lower LAS-r (37.3% vs. 42.3%, *p* < 0.05), and lower LAS-cd (22.9% vs. 28.4%, *p* < 0.05) (Figure 2). LAS-ct did not differ significantly (14.8% vs. 13.8%, *p* = 0.38) (Table 2, section C).

Receiver operating characteristic (ROC) analysis was performed for LAS-r, LAS-cd, and the LA stiffness index. LAS-cd showed the highest AUC (0.711; 95% CI 0.608–0.813; *p* < 0.001) (Figure 3). LAS-cd showed the highest AUC (0.711; 95% CI 0.608–0.813; *p* < 0.001) (Figure 3). Overall, the ROC analysis indicated modest discriminative ability of the evaluated LA functional parameters.

Subgroup analysis divided ESUS patients into those with confirmed PFO (*n* = 25) and those without interatrial shunting (*n* = 25). One-way ANOVA and Kruskal–Wallis tests revealed significant between-group differences regarding diastolic function (E wave: F = 4.253, *p* = 0.017; lateral e’: F = 4.439, *p* = 0.014; septal e’: F = 8.723, *p* < 0.001), LA strain (LAS-r: H(2) = 8.968, *p* = 0.011; LAS-cd: F = 6.805, *p* = 0.002), and LA stiffness index (F = 3.391, *p* = 0.038) (Table 3, sections A and B).

Post hoc Tukey HSD analysis showed that the control group had significantly higher E wave (*p* = 0.040) and septal e’ (*p* = 0.002) velocities compared to patients with PFO. Lateral e’ velocity was significantly higher (*p* = 0.015), and LA stiffness index was lower (*p* = 0.041) in the control group compared to stroke patients without PFO. LAS-cd was significantly higher in controls than in both ESUS subgroups (*p* = 0.008 vs. PFO; *p* = 0.011 vs. non-PFO).

The post hoc analysis for LAS-r, performed with the Kruskal–Wallis test, revealed statistically significant differences between the control group and the group of patients with a history of stroke and confirmed PFO (*p* < 0.033), as well as between the control group and the group of stroke patients without PFO (*p* < 0.007). No statistically significant differences were found between the two stroke subgroups (with PFO vs. without PFO).

## 4. Discussion

### 4.1. Altered Left Atrial Function in ESUS Patients: Clinical Relevance and Comparison with Existing Evidence

In our study, significant differences in LA function were observed between patients with ESUS and healthy controls. In this context, the evaluation of LA function is gaining increasing importance in clinical practice. Echocardiography, being widely available, is an indispensable diagnostic tool: it enables the assessment and early detection of subtle changes indicative of LA dysfunction [16,17]. LAS is being increasingly adopted in clinical practice due to its sensitivity and reproducibility in evaluating atrial function [18]. In patients with heart failure with preserved ejection fraction (HFpEF), reduced LA compliance has been associated with elevated cardiovascular risk [19].

Moreover, decreased LAS-r values have been confirmed to correlate with thromboembolic complications and new-onset AF, particularly in individuals with cardiac amyloidosis and mitral stenosis [20,21,22]. In a study by Olsen et al., both reservoir and contraction phase LAS independently predicted subclinical AF in older adults at high risk of ischemic stroke [18].

In patients with a history of ischemic stroke, LA strain analysis also serves as a valuable adjunct in clarifying stroke etiology. Only a limited number of studies have compared left atrial functional parameters between patients with ESUS and control subjects. Bashir et al. demonstrated that individuals with ESUS had significantly lower LAS values compared to those with non-cardioembolic strokes [23]. These findings are further supported by the existing literature. Alhakak et al. identified reduced peak atrial longitudinal strain (PALS) as a significant predictor of AF [24]. A 2024 meta-analysis by Clark et al. confirmed these observations, highlighting altered LA metrics in the ESUS population [25].

In 2022, Sade et al. demonstrated that patients with ESUS exhibited significantly lower LAS values during both the reservoir and contraction phases compared to healthy volunteers (LAS-r: 21.4 ± 6.1% vs. 40.2 ± 6.7%, *p* < 0.0001; LAS-ct: 13.3 ± 3.8% vs. 19.6 ± 4.9%, *p* < 0.0001) [26]. Similarly, Zhang et al. reported significantly reduced LAS-r and LAS-cd in patients with a history of ESUS compared to healthy controls (LAS-r: 27.8 ± 7.1% vs. 37.6 ± 7.2%, *p* < 0.001; LAS-cd: 14.3 ± 3.8% vs. 21.5 ± 7.9%, *p* < 0.001) [27]. Overall, our findings are consistent with previous studies showing impaired LA function in patients with ESUS, although some differences in individual LA strain parameters were observed.

In our study, significant differences were observed between the analyzed groups in echocardiographic parameters used to assess left ventricular (LV) diastolic function. Patients with ESUS demonstrated statistically significantly lower values of E-wave velocity, E/A ratio, and early diastolic mitral annular velocity (e’), measured medially and laterally by tissue Doppler imaging. However, all absolute values remained within the generally accepted reference ranges in both cohorts.

Additional analysis revealed that ESUS patients exhibited reduced LAS during the reservoir and conduit phases, and increased strain during the booster pump phase. Among these, LAS-r and LAS-cd differed significantly between groups. Moreover, the LA stiffness index—derived from LV diastolic function and LA contractile performance—was significantly elevated in the ESUS group.

Although LAS-cd showed the highest discriminative ability (AUC = 0.711), the overall discriminative performance of the evaluated LA functional parameters was modest. Therefore, these findings should be considered exploratory and do not support the use of these parameters as stand-alone diagnostic or risk-stratification tools.

### 4.2. Impact of PFO on Atrial Function: Insights from Subgroup Analysis

Data on the impact of PFO on LA dysfunction are also limited. In the study by Gazagnes et al. (2020), no significant differences were observed in LAS between patients with PFO and those without PFO [28]. Our subgroup analysis did not demonstrate a consistent association between the presence of a PFO and LA functional impairment in the ESUS population. This may suggest that LA dysfunction can coexist with PFO and may represent another potential mechanism of stroke in these patients.

Despite the absence of overt clinical or imaging-based evidence of diastolic dysfunction, these findings may suggest differences in LV myocardial compliance between the groups. However, the most prominent findings pertain to LA function, as assessed through strain analysis. These differences may represent secondary changes associated with subclinical LV dysfunction or primary structural and functional remodeling of the atrial wall.

## 5. Limitations

This study has certain limitations that should be acknowledged. The relatively small sample size and the exploratory nature of the statistical analyses may affect the interpretation of our findings, particularly in the subgroup analyses. Therefore, the results should be interpreted with caution and require confirmation in larger studies.

Although all ESUS patients underwent 72 h Holter monitoring and no AF or atrial flutter was detected, prolonged rhythm monitoring was not systematically performed. Therefore, occult low-burden paroxysmal AF cannot be excluded in some patients and may have contributed to the observed LA functional abnormalities. Future studies incorporating prolonged or continuous rhythm monitoring are needed to clarify the relationship between impaired LA strain, atrial cardiomyopathy, and subsequent AF detection.

Detailed data on cardiovascular risk factors, renal function, and medication use were not systematically collected in both groups, precluding comprehensive between-group comparisons and multivariable adjustment for these potential confounders. Therefore, residual confounding by factors that may independently affect LA mechanics and LV diastolic function cannot be excluded.

Furthermore, echocardiography was performed 1–3 weeks after the index stroke. Acute or subacute stroke-related alterations in autonomic regulation, loading conditions, and hemodynamic status may potentially influence LA strain and LV diastolic parameters. Therefore, the cross-sectional nature of the echocardiographic assessment does not allow us to determine whether the observed LA dysfunction preceded the stroke or was, at least partly, influenced by the cerebrovascular event.

As all LA strain measurements were performed by a single observer, inter-observer reproducibility was not assessed.

The inclusion criteria required transesophageal and transthoracic echocardiography with speckle-tracking analysis to be performed within 1–3 weeks after the stroke, which in itself excluded patients with severe neurological deficits who could not safely undergo the examination. This may have introduced selection bias and limits the generalizability of the results to the broader ESUS population. Moreover, echocardiographic assessments were performed at a single time point, preventing evaluation of longitudinal changes in atrial function after stroke. Future research involving larger cohorts and repeated follow-up imaging is warranted to better characterize the temporal dynamics and clinical significance of atrial dysfunction in ESUS.

## 6. Conclusions

Patients with ESUS demonstrated impaired LA reservoir and conduit function despite preserved LA size and volume compared with age- and sex-matched healthy controls. These findings indicate an association between ESUS and subclinical LA functional abnormalities. LA strain may provide complementary information on atrial function beyond conventional measures of LA size; however, its clinical and prognostic significance in ESUS requires confirmation in larger prospective studies with prolonged rhythm monitoring and longitudinal follow-up.

## Figures and Tables

**Figure 1:**
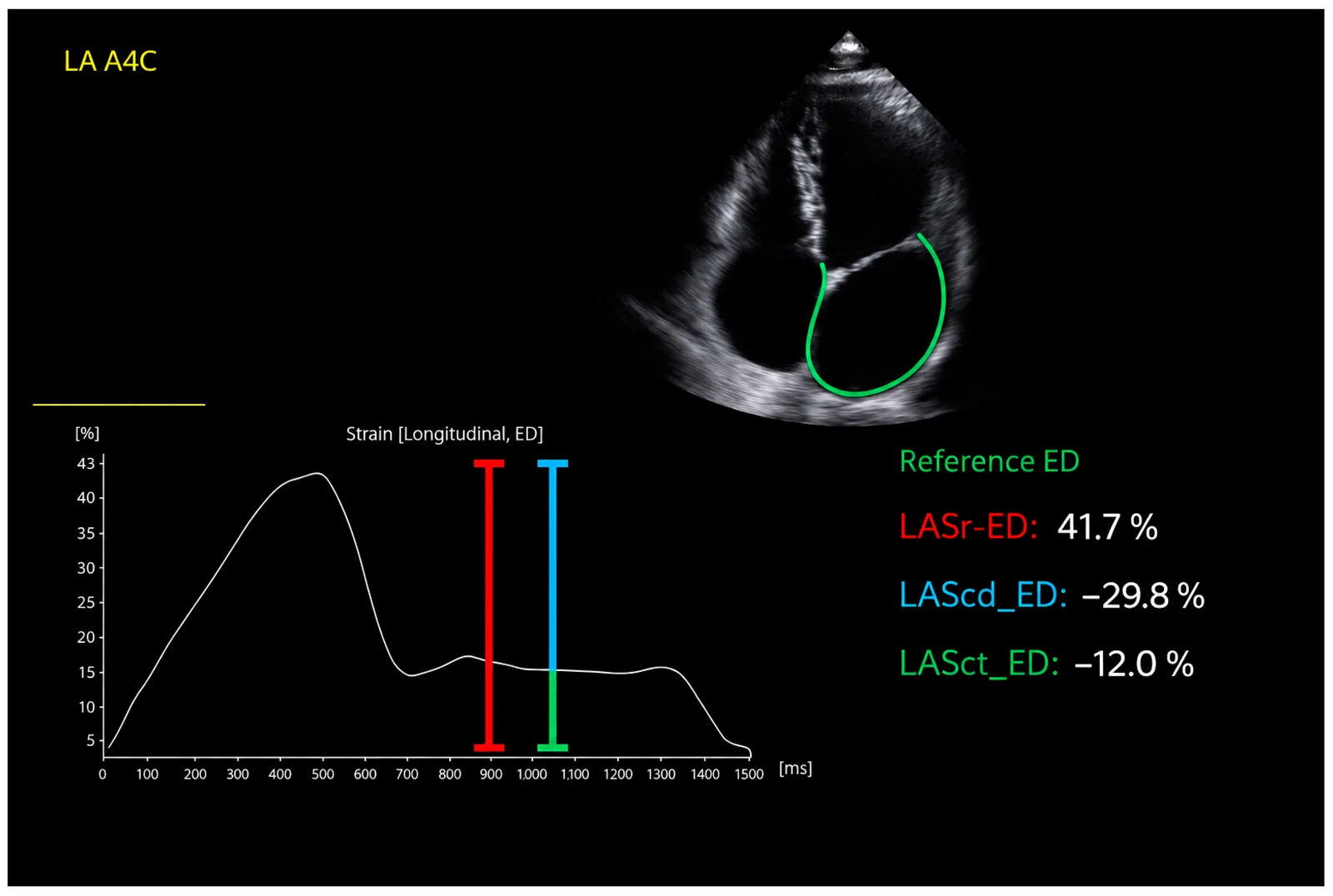
Echocardiographic assessment of left atrial strain. Speckle tracking echocardiographic assessment of left atrial strain in the apical four-chamber (A4C) view: the reservoir phase (LAS-r, red line), the conduit phase (LAS-cd, blue line), and the contraction phase (LAS-ct, green line). The green contour indicates the tracked left atrial endocardial border used for strain analysis.

**Figure 2:**
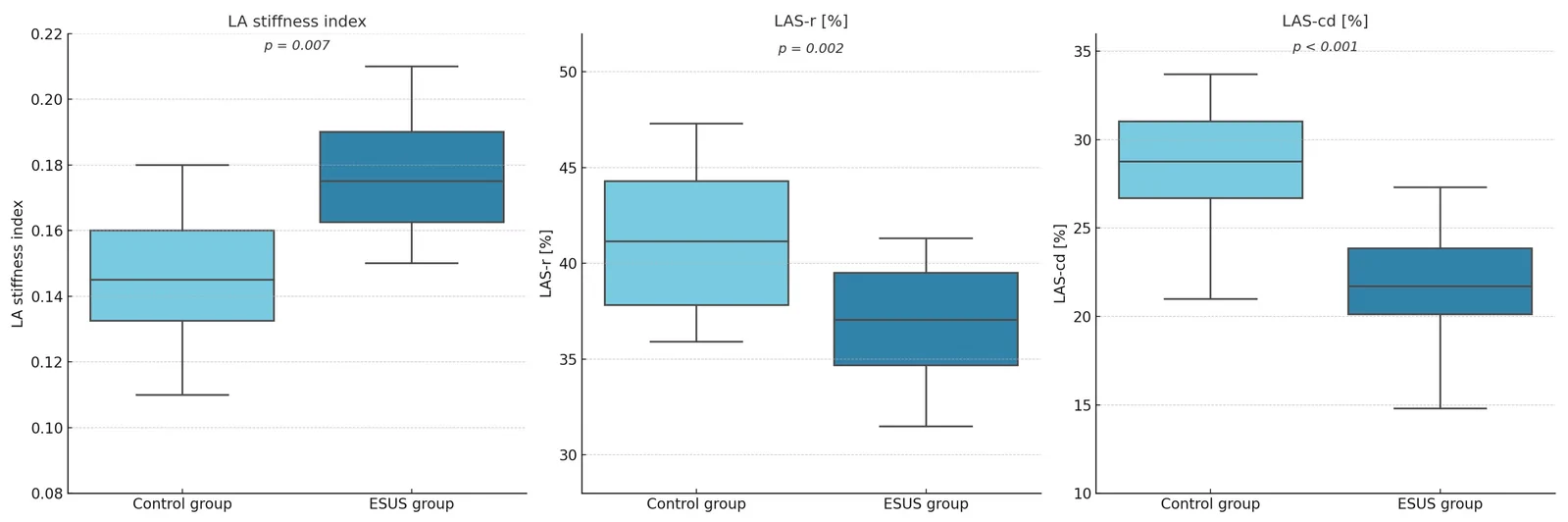
Box plots. Box plots demonstrating statistically significant differences in the left atrial stiffness index and left atrial strain parameters during the reservoir phase (LAS-r) and conduit phase (LAS-cd) between patients with a history of embolic stroke of undetermined source (ESUS group) and the control group.

**Figure 3:**
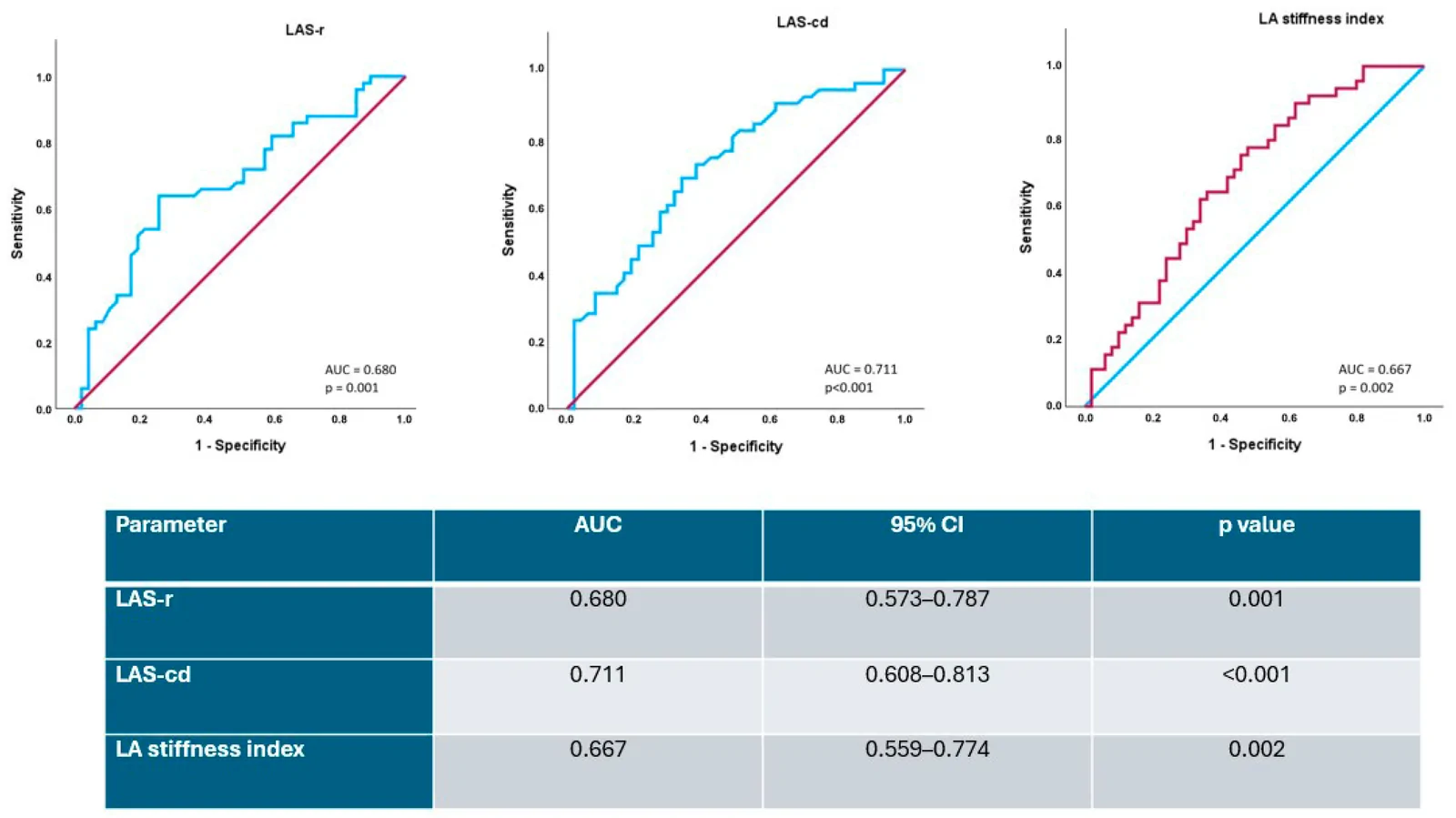
ROC curves. ROC curves showing diagnostic performance of LA functional parameters for differentiating ESUS patients from healthy controls. The colored stepwise lines represent the ROC curves, while the diagonal lines represent the reference lines of no discrimination.

**Table 1:** Comparison of anthropometric parameters between patients with a history of ischemic stroke and the control group. Variables with a normal distribution are presented as mean ± standard deviation, while those with a non-normal distribution are expressed as median and interquartile range (Q1–Q3).

	Patients with Stroke History (*n* = 50)	Control Group (*n* = 50)	*p* Value
Age (years)	42 Q1 36.00, Q3 50.00	42 Q1 33.75; Q3 47.25	0.35
Weight (kg)	74.72 ± 15.88	72.96 ± 13.45	0.55
Height (cm)	168 Q1 164, Q3 176	168 Q1 164, Q3 176	0.90
BMI (kg/m^2^)	25.67 Q1 22.22, Q3 29.62	25.1 Q1 23.10, Q3 27.53	0.52
BSA (m^2^)	1.85 ± 0.22	1.84 ± 0.18	0.70

Regarding cardiovascular risk factors in the ESUS group, 11 patients (22%) had arterial hypertension, one patient (2%) had impaired fasting glucose, and two patients (4%) were active smokers. None of the patients had diabetes mellitus or known chronic kidney disease.

**Table 2:** Comparison of echocardiographic parameters between patients with a history of embolic stroke of undetermined source (Patients with stroke history) and the control group. Variables with a normal distribution are presented as mean ± standard deviation, while those with a non-normal distribution are expressed as median and interquartile range (Q1–Q3).

Parameter	Patients with Stroke History (*n* = 50)	Control Group (*n* = 50)	*p* Value
**Section A—**parameters related to left ventricular size and function			
LVEF [%]	64.87 ± 4.43	64.79 ± 4.21	0.93
IVS [mm]	9.63 Q1 8.57, Q3 10.40	9.11 Q1 8.46, Q3 10.10	0.28
LV [mm]	46.52 ± 4.91	46.95 ± 3.65	0.63
PW [mm]	8.83 ± 0.90	8.68 ± 1.08	0.46
LV mass [g]	147.32 ± 38.51	145.02 ± 33.29	0.75
LV mass index [g/m^2^]	78.00 Q1 69, Q3 85	79.50 Q1 70, Q3 87	0.58
E wave [cm/s]	76.50 ± 13.21	84.84 ± 15.54	0.005
A wave [cm/s]	69.26 ± 13.1	68.13 ± 13.01	0.69
E/A	1.11 Q1 0.89, Q3 1.35	1.24 Q1 1.08, Q3 1.44	0.024
Dec T [ms]	180.50 ± 34.77	172.12 ± 33.54	0.33
e’ sept [cm/s]	10.3 Q1 8.54, Q3 11.70	12.15 Q1 10.78, Q3 12.90	<0.001
e’ lat [cm/s]	14.28 ± 2.92	16.03 ± 3.08	0.005
E/e’	6.03 Q1 5.60, Q3 7.01	5.82 Q1 5.23, Q3 6.99	0.24
TR Vmax [m/s]	2.03 ± 0.28	2.08 ± 0.21	0.35
**Section B—**measurements and volumes of the left atrium			
LA [mm]	34.54 ± 4.65	35.04 ± 3.64	0.52
LAA [cm^2^]	17.39 ± 2.89	17.79 ± 2.33	0.45
LAV [mL]	44.45 ± 12.60	46.74 ± 10.23	0.32
LAVI [mL/m^2^]	24.00 ± 5.41	25.53 ± 5.14	0.15
**Section C—**left atrial strain parameters			
LAS-r [%]	37.30 Q1 33.10, Q3 41.30	42.30 Q1 36.18, Q3 47.33	0.002
LAS-cd [%]	22.89 ± 7.06	28.35 ± 7.25	<0.001
LAS-ct [%]	14.84 ± 5.86	13.80 ± 5.82	0.38
LA stiffness index	0.171 ± 0.037	0.149 ± 0.039	0.007

**Table 3:** Comparison of echocardiographic parameters among patients with stroke history and patent foramen ovale (PFO), patients with stroke history without PFO, and the control group. Variables with a normal distribution are presented as mean ± standard deviation, while non-normally distributed variables are expressed as median and interquartile range (Q1–Q3).

Parameter	Patients with Stroke History and PFO (*n* = 25)	Patients with Stroke History Without PFO (*n* = 25)	Control Group (*n* = 50)	*p*-Value
**Section A—**parameters related to left ventricular size and function				
LVEF [%]	66.25 ± 4.44	63.70 ± 4.34	64.59 ± 3.91	0.08
IVS [mm]	9.38 Q1 9.01, Q3 10.40	9.79 Q1 8.65, Q3 10.25	9.00 Q1 7.81, Q3 9.48	0.45
LV [mm]	46.60 ± 5.10	47.05 ± 4.93	44.88 ± 4.09	0.87
PW [mm]	8.84 ± 0.67	8.80 ± 1.06	8.58 ± 1.31	0.73
LV mass [g]	146.65 ± 30.80	149.17 ± 39.95	135.88 ± 39.22	0.91
LV mass index [g/m^2^]	77.00 Q1 70.00, Q3 87.00	79.00 Q1 70.50, Q3 84.00	73.00 Q1 64.00, Q3 87.00	0.94
E [cm/s]	74.94 ± 9.03	76.76 ± 14.27	89.11 ± 14.02	0.017
A [cm/s]	65.50 Q1 54.40, Q3 78.30	68.50 Q1 63.90, Q3 74.10	69.50 Q1 63.80, Q3 75.00	0.60
E/A	1.12 Q1 0.97, Q3 1.38	1.11 Q1 0.88, Q3 1.33	1.25 Q1 1.17, Q3 1.44	0.07
Dec T [ms]	195.00, Q1 161.00, Q3 210.00	173.00, Q1 157.00, Q3 186.00	169.00, Q1 152.00, Q3 190.00	0.47
e’ sept [cm/s]	10.18 ± 1.85	10.52 ± 1.98	12.44 ± 2.59	<0.001
e’ lat [cm/s]	14.21 ± 2.55	13.88 ± 2.79	16.39 ± 2.94	0.014
E/e’	6.30 ± 1.19	6.37 ± 0.99	6.30 ± 1.18	0.66
TR Vmax [m/s]	2.04 ± 0.32	2.02 ± 0.28	2.10 ± 0.22	0.37
**Section B—**left atrial strain parameters				
LAS-r [%]	38.20 Q1 32.90, Q3 42.00	36.65 Q1 33.45, Q3 40.25	42.10 Q1 36.30, Q3 48.90	0.011
LAS-cd [%]	22.59 ± 6.37	22.98 ± 8.00	29.88 ± 7.18	0.002
LAS-ct [%]	15.14 ± 5.75	15.33 ± 6.27	13.39 ± 5.68	0.66
LA stiffness index	0.174 ± 0.046	0.170 ± 0.033	0.151 ± 0.042	0.038

## Data Availability

The data presented in this study are available from the corresponding author upon reasonable request. The data are not publicly available due to privacy and ethical restrictions.

## Abbreviations

AUC	area under the curve
CI	confidence interval
LAS-r	left atrial reservoir strain
LAS-cd	left atrial conduit strain
LA stiffness index	left atrial stiffness index
LVEF	left ventricular ejection fraction
IVS	interventricular septum thickness
LV	left ventricular end-diastolic diameter
PW	posterior wall thickness
E wave	early diastolic transmitral flow velocity
A wave	late diastolic transmitral flow velocity
Dec T	E-wave deceleration time
e′ sept	septal mitral annular early diastolic velocity
e′ lat	lateral mitral annular early diastolic velocity
TR Vmax	maximal tricuspid regurgitation velocity
LA	left atrial anteroposterior diameter
LAA	left atrial area
LA vol	left atrial volume
LAVI	left atrial volume index
LAS-ct	left atrial contraction strain
